# Evaluation of a 3D‐Printed Model as Complete Case Scenario in Undergraduate Dental Education—Diagnosis, Treatment Planning and Clinical Practice

**DOI:** 10.1111/eje.13100

**Published:** 2025-04-25

**Authors:** Sebastian Bürklein, Edgar Schäfer, David Donnermeyer

**Affiliations:** ^1^ Central Interdisciplinary Ambulance in the School of Dentistry University of Münster Münster Germany; ^2^ Department of Restorative, Preventive and Pediatric Dentistry, ZMK Bern University of Bern Bern Switzerland

**Keywords:** 3D printing, dental education, dental students, digital learning, digital technology, simulation training, universal digital dental models

## Abstract

**Introduction:**

3D‐printed teeth/models are important adjuncts in dental education. Nevertheless, there is a lack of simulated cases to learn and understand complex treatment scenarios, including anamnesis, diagnosis, treatment planning and therapy.

**Material and Methods:**

Third‐year students (*n* = 44) received a complete 3D‐printed model (upper and lower jaw) of a patient who needed emergency treatment. Based on the information provided (i) general history, (ii) specific dental history, (iii) radiographs, the students made a diagnosis and planned the treatment, which they performed independently under supervision. The case included periodontal, restorative, endodontic and surgical treatments and semi‐permanent splinting. Using a 3‐ or 5‐point Likert scale, students rated each treatment episode, the learning outcome and the impact of the model on training using a questionnaire. Chi‐square test served for statistical analysis.

**Results:**

The majority (63.6%) stated that the interdisciplinary model allowed a better learning effect than the approach dealing with each section separately (*p* < 0.05). Almost all students rated the diagnostic process as excellent (88.0%–95.5%), as the general history, dental history and radiographs were consistent with the clinical findings. Periodontal therapy was rated significantly lower compared to all other areas (*p* < 0.05). For endodontic treatment, the students disagreed, abstaining from practising on extracted human teeth. However, the students felt confident to perform all treatment steps in the following clinical courses.

**Conclusion:**

Customised, interdisciplinary 3D‐printed teaching models covering complex treatment strategies were best suited to enhance dental students' skills and foster their enthusiasm for the integrated diagnosis and treatment planning process. Their implementation into dental education is strongly recommended to improve both training and future patient care.

## 1 Introduction

The history of the development of 3D‐printing can be traced back to the 1980s, when the first 3D‐printing technologies were invented. Hideo Kodama was the first who developed rapid prototyping layer‐by‐layer using photo‐polymerisation, representing the precursor of stereolithography (SLA) [1]. Due to a formal error by Kodama when filing the patent, Charles Hull is considered the inventor and namesake of stereolithography. In 1987, 3D Systems (Rock Hill, SC, USA) launched the first 3D‐printer, the SLA‐1 stereolithographic printer [2].

Nowadays, 3D‐printing is well established in dentistry, especially in the production of removable denture bases/full and partial dentures, try‐in devices, long‐term temporaries, indirect bonding trays for orthodontic applications, splints, prosthodontic or orthodontic models, custom trays, gingiva masks for implant models and surgical guides [3, 4, 5, 6]. In general, the use of 3D‐printing in dentistry still continues to grow, with researchers and dental professionals exploring new applications for the technology [7, 8]. Today, 3D‐printing is widely used in dental education, with dental schools around the world incorporating the technology into their curricula. As technology continues to advance, it is likely that there will be even more innovative applications of 3D‐printing in dental education and in patient care and treatment planning [9].

Recently, the number of publications of 3D‐printed models in dental education has increased significantly [9]. Nevertheless, most programs in undergraduate education do not cover interdisciplinary approaches by simulating complex treatments addressing all fields of dentistry simultaneously. Commercially available teaching models were developed for singular fields of dentistry (e.g., prosthodontic treatment, periodontology and trauma) due to practicability. Numerous available studies that evaluated 3D‐printed models were also designed for special treatment steps, for example, excavation and preparation [10, 11, 12], pulp‐capping [13], build‐up and crown/inlay‐preparation [14, 15, 16, 17, 18, 19], dentin post preparation [20], prosthodontics [21], surgery [22, 23, 24, 25], apicoectomy [26], paediatric dentistry [27], dental traumatology [28] and endodontics [29, 30].

However, there are no studies available using interdisciplinary modular 3D‐printed models for all steps of dental treatment including diagnosis, treatment planning and the clinical management representing complete and complex treatment cases. Thus, the aim of this study was to evaluate the implementation of an individual modular 3D‐printed model as a complete treatment case and to evaluate its suitability as a component of the undergraduate curriculum. The null hypothesis was that students would assess the new model equally as currently available models that are only appropriate for one discipline, simply focusing on replicating specific treatment steps.

## 2 Material and Methods

### 2.1 Institutional Review Board Clearance

The local ‘Quality Improvement Commission’ approved the project (QV1249). The basis for this funding is the federal Study Quality Act, as it is demanded by the ‘Council of Science and Humanities’. The aim of the fund is to improve teaching and study conditions. This commission consists of both academic and student members. Thus, an ethical vote was not applicable due to the approval process by students, teachers and federal government. Additionally, this kind of funding requires an evaluation of the implemented intervention/task to justify the money transfer.

All 3rd year students participated in this project after successfully passing the class. The questionnaire was completely anonymised and no personal data were assessed to identify any student. No personal information other than gender, age and educational status were gathered from all participants. Neither the decision to participate or not, nor the results of the questionnaire had any consequence on a student's academic progress. Student ratings could not be linked to individual course grades or academic performance. Data were processed and stored in accordance with current data protection laws.

### 2.2 Conception of Models, Choice of Printers and Materials Used

According to the method described by Hanafi et al., a 3D radiograph (CBCT) of a Thiel‐fixated human skull provided by the institute of anatomy and a selection of extracted human teeth, a printable STL dataset was created. The model presented the entire dentition with the periodontal ligament and the corresponding surrounding tissues. The free medical software InVesaliusTM (CTI Open Labs Renato Archer, Campinas, Sao Paulo, Brazil) served for reconstruction and conversion of the DICOM data to STL data sets, and Geomagic FreeformTM (3D Systems Inc., Rock Hill, South Carolina, USA) was used for the design of its specifications. The specimens were printed with the stereolithographic XFAB 2500SD SLA 3D‐Printer and the associated dental model resin (model‐basis: DSW Invicta 917; teeth: Temporis; (all: DWS, Thiene, Italy)).

#### 2.2.1 Modell Specifications

**FIGURE 1:**
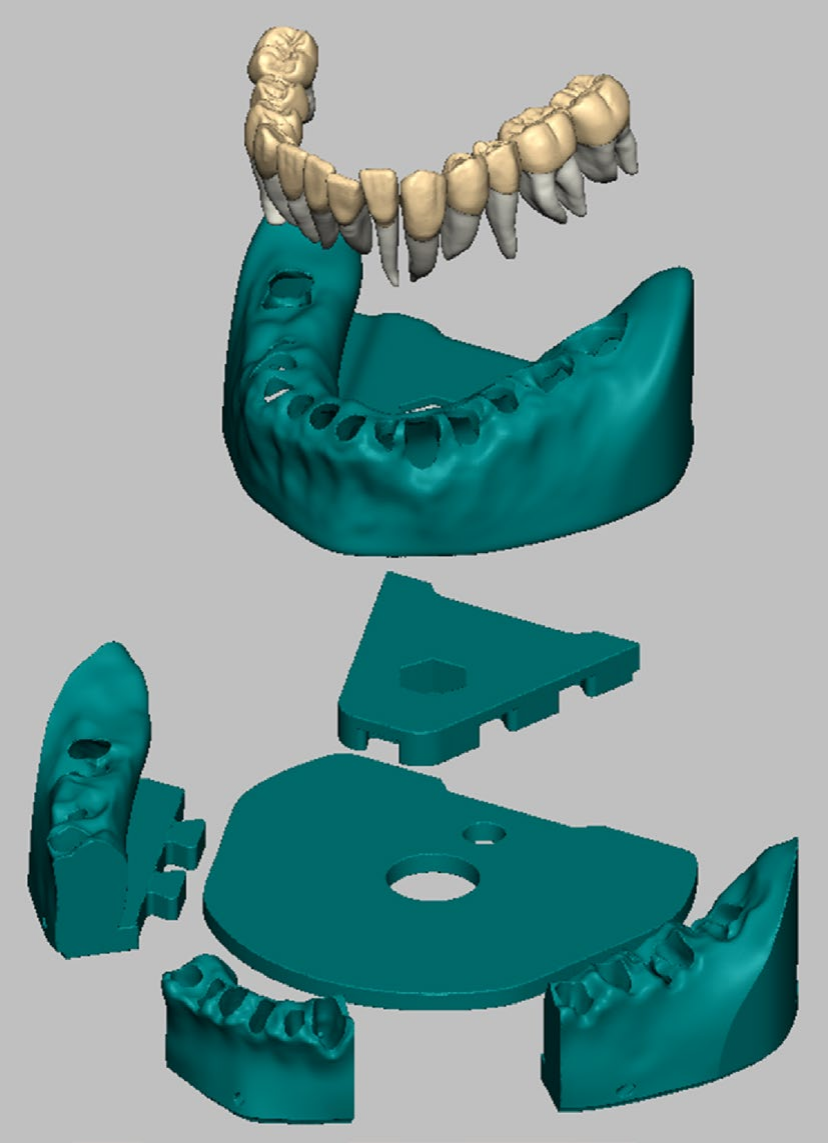
Construction details of the universal 3D‐printed model.

**FIGURE 2:**
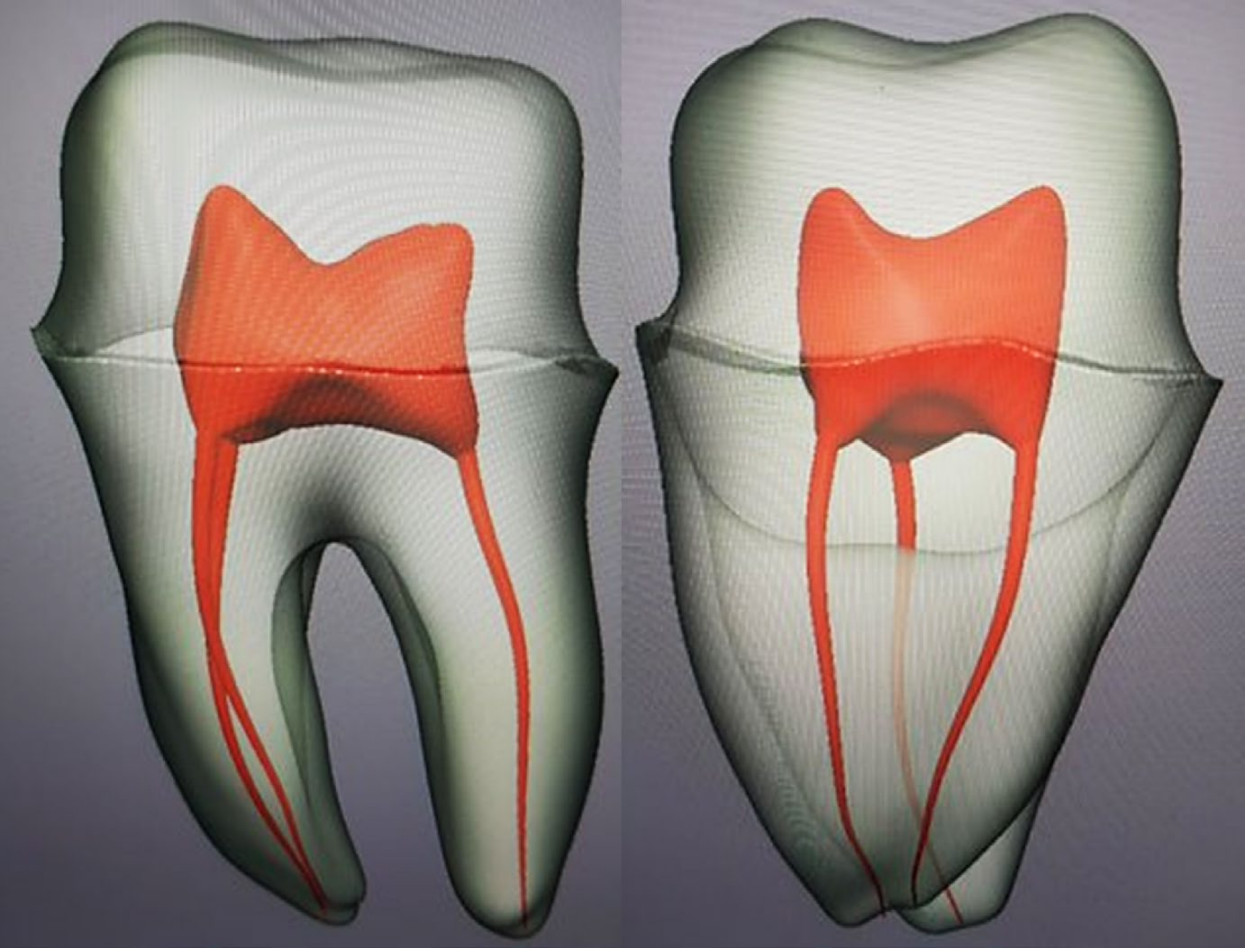
Root canal system. Lateral and mesial view of the root canal system under construction.

The lower and upper jaw were printed in sextants (Figures 1 and 2). A central plate provided the fixation of all sextants on the base plate. The teeth were fabricated with one or more circumferential prominent rings around the middle and/or apical part of the roots acting as a patrixes with matching matrixes in their corresponding sockets to ensure a tight fit (Figure 2). An individual gingival mask was designed and made from a silicone (Replisil FB 25, Siliconic, Lonsee, Germany) using an individual mould.

**FIGURE 3:**
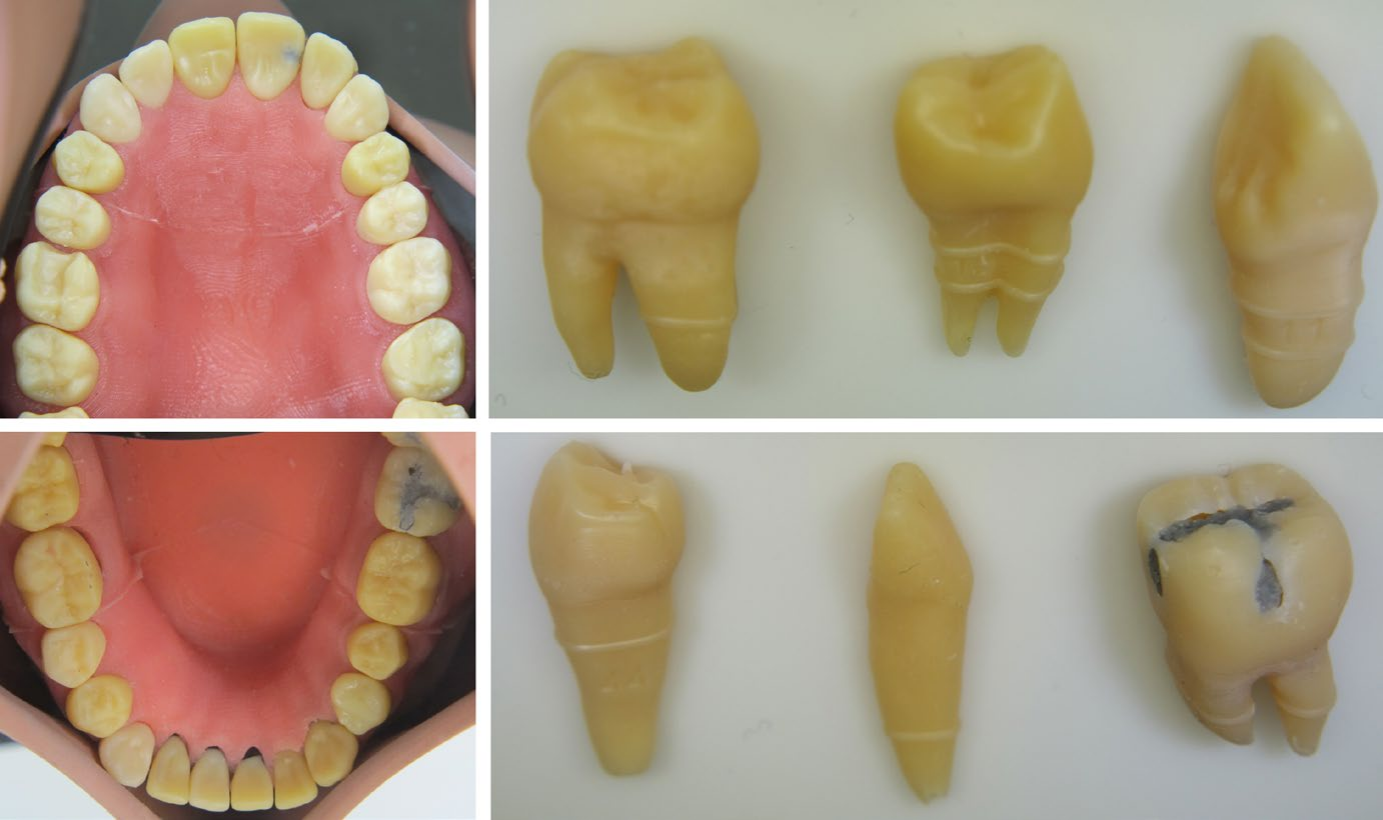
Occlusal view of the printed model with gum mask in the phantom heads. Exemplary teeth with their unique properties, for example, Carious lesion.

The teeth that received root canal treatment were made in two parts (Figure 3). First, the tooth without the associated crown was printed with the inherent root canal system, and thereafter the root canals were checked for patency after washout of the residual resin with a centrifuge. Subsequently, the root canal system was filled with red‐coloured hydrogel, and the corresponding crown was adhesively cemented in place.

**FIGURE 4:**
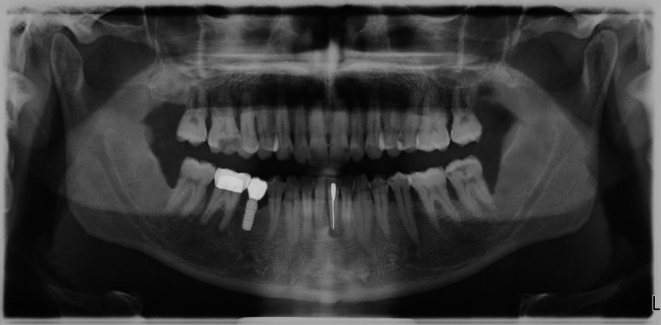
Panoramic view of the patient/case with all simulated findings (periodontitis; apical periodontitis 46; caries 21, 25, 37; lost restoration 16; osteolysis due to vertical root fracture tooth 31.

**FIGURE 5:**
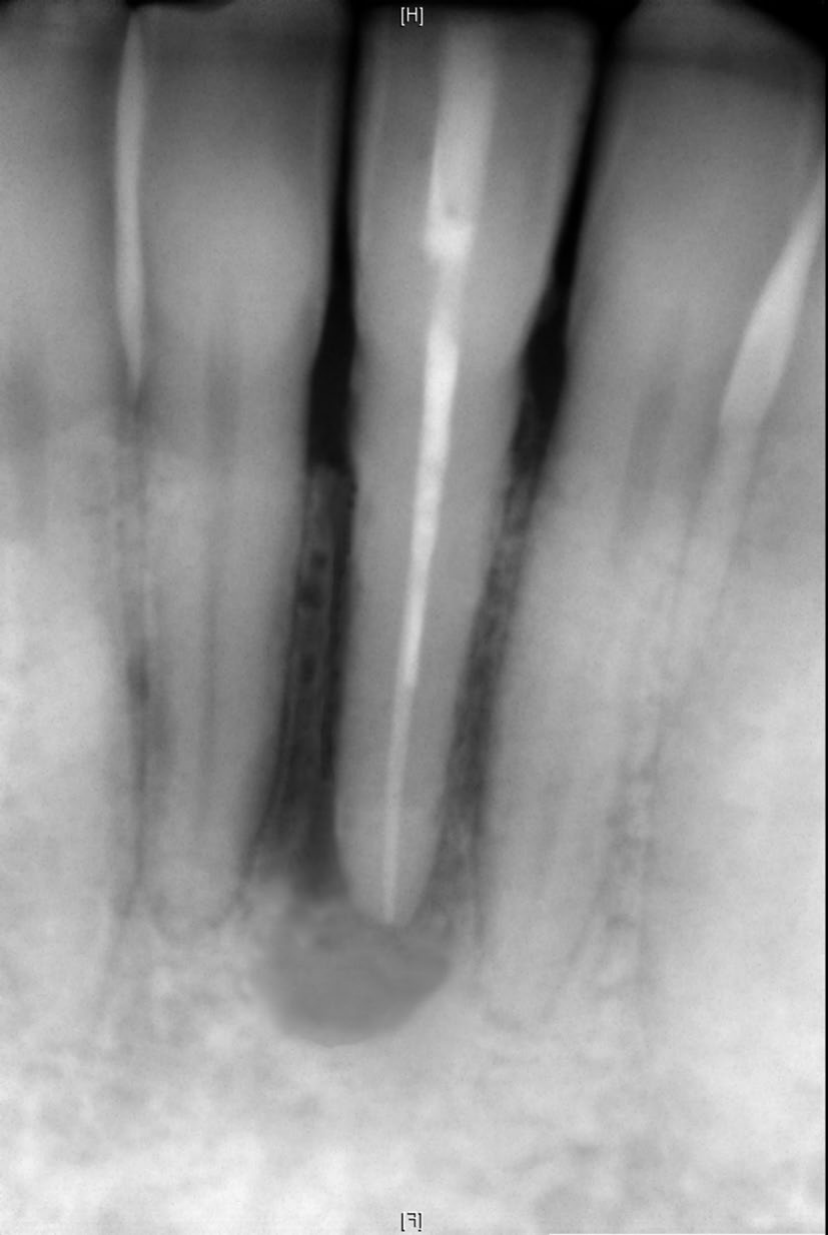
Intraoral radiograph of the anterior mandibular region. Tooth 31 shows a typical lateral J‐halo lesion due to a vertical root fracture.

After successfully passing the phantom course in the 3rd year of the dental curriculum, the 3D‐printed models were handed out to the students (*n* = 44) as a complete and complex patient case including medical history, general and special dental anamnesis and corresponding radiographs (Figures 4 and 5). The students received all the necessary information to be able to carry out all steps comparable to a situation in clinical practice: Diagnosis, treatment planning and the conduct of the treatment. All treatments met the current level of technical skills and knowledge of the students according to the regular training protocol.

The following data concerning the patient were provided: Gender = male, age = 52 years, smoker > 10 cigarettes/day, Hypertension (controlled by medication), drugs = Nifedipine.

Special anamnesis:

- Dragging toothache in the region of the left upper and lower jaw on ‘hot and cold’ stimuli.
- Moderate bite‐to‐bite discomfort in the right lower jaw.
- Upper front: roughness perceptible with the tongue.
- A few weeks ago, the patient had lost a gold cast restoration in the right upper jaw. Response to sensitivity test = positive, no tenderness to percussion, actually no pain.
- At present, biting hard food off with the incisor teeth is impossible as the patient described that one of the mandibular incisors is mobile. Dislocation is associated with pain. Occasionally, putrid fluid drains from the sulcus. The tooth has already been root canal‐treated some years ago.
- Furthermore, a regular gum bleeding and a foetor ex ore were described.
- A periodontal therapy was performed more than 5 years ago. At that time, periodontal pockets up to 7 mm had been measured.
- The wisdom teeth had been removed a long time ago.

In addition, radiographs exactly adapted to the case were provided (panoramic view and periapical radiographs of the anterior region of the lower jaw).

The simulated findings represented the following diagnoses:

- Lost inlay tooth 16 due to secondary decay
- Carious lesions: tooth 21 (distal‐palatal), tooth 25 (occlusal‐mesial), tooth 37 (mesial‐occlusal‐buccal)
- Apical periodontitis: tooth 46
- Periodontitis: generalised stage III, grade C; medication‐associated gingiva hyperplasia
- Vertical root fracture of tooth 31

The students' task was to achieve appropriate diagnoses with the provided data, to develop a treatment plan, and then to carry out the adequate therapy independently. The treatment plan was first presented to the teachers, discussed and approved if completed.

The treatment plan should ideally include the following treatment procedures:

- Root canal treatment: tooth 46
- Direct composite restorations/fillings: teeth 21,25,37
- Temporary restoration: tooth 16
- Periodontal therapy (to perform steps 0–2, that is, supra‐ and sub‐gingival removal of biofilm/calculus, to plan step 3 and 4 with the aftercare and supportive periodontal therapy (=SPT) – here SPT interval 3–6 months up to 2 years).
- Consultation with general practitioner (antihypertensive drug associated hyperplasia of the gingiva)
- Extraction, extra‐oral shortening and semi‐permanent splinting as an immediate treatment: tooth 31
- Preparation of a ceramic partial crown: tooth 16

**FIGURE 6:**
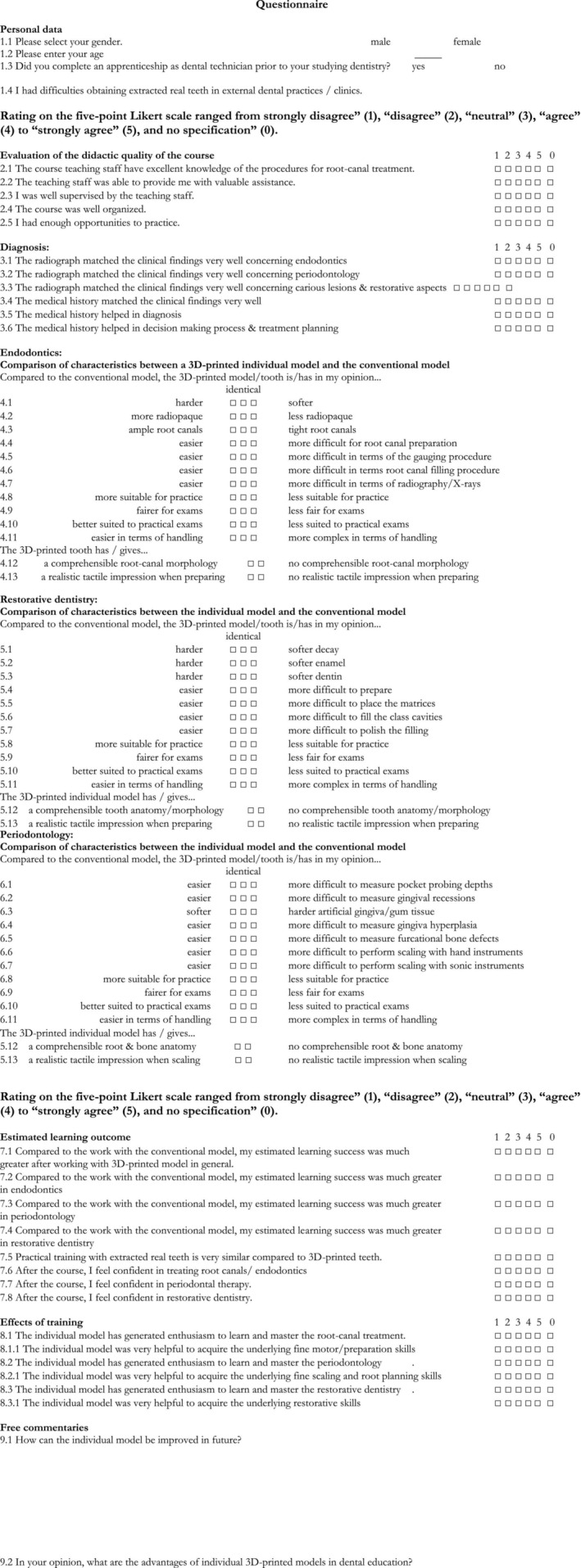
Questionnaire for the evaluation of the teaching module and the 3D‐printed model by the students.

After finishing the complete treatment that was performed under supervision by the teaching staff identically to the clinical courses when treating real patients, the students evaluated the teaching module and the 3D‐printed model using an anonymized questionnaire (Figure 6).

For (quantitative and qualitative) control of the periodontal treatment (scaling and root planning), all roots were painted black prior to their fixation in the model.

### 2.3 Time Management

Students had no time limitation for execution of all tasks. Nonetheless, the pilot project was planned within 1 week of the regular time allocated in the regular curriculum. Educators calculated 12 h for the complete procedures according to the skills level of the students and gave four extra hours to guarantee that all students were able to finish the treatment in this pilot project.

### 2.4 Student Questionnaire on the Satisfaction/Relevance of the Model

The questionnaire was adopted from already established and validated questionnaires and adapted to the relevant settings, that is, the multidisciplinary approach by addressing all disciplines separately [14, 15, 16, 17, 19].

Additionally, the questionnaire was validated using a pre‐test in a cohort of ten 4th year students that had already finished the corresponding course and performed all treatment steps of the model in advance. They conducted the questionnaire twice within an interval of 2 weeks, and the reliability (Cronbach's alpha) was 0.89.

#### 2.4.1 Survey Questionnaire

The questionnaire consisted of (1) socio‐demographic data (first four questions of the questionnaire); (2) participants' perception of the entire course (questions 2.1–2.5); (3) participants' perception of the 3D‐printed model matching to clinical, radiographic and medical history (questions 3.1–3.6); (4) participants' perception of endodontic findings and treatment (question 4.1–4.13); (5) participants' perception of periodontal findings and treatment (question 5.1–5.13); (6) participants' perception of restorative findings and treatment (question 6.1–6.13); (7) participants' perception of estimated learning outcome of the 3D‐printed universal teaching model (question 7.1–7.8); (8) participants' estimated training effects of its use in terms of being confident in future treatments (question 8.1–8.3.1); (9) free comments (Figure 6).

Items 3, 4 and 5 were identically structured to allow an interdisciplinary comparison. All items were represented by multiple‐choice questions. The estimated time needed to fill in the questionnaire was about 10 min.

The Chi‐square test was used for statistical analysis and the significance level was set at *p* < 0.05.

## 3 Results

### 3.1 Demographic Data

All students performed the treatment of the simulated and 3D‐printed case. The majority of the 3rd year students (23.2 ± 3.6 years) were females (70.5%). 1/3 of the students reported some difficulties in collecting extracted human teeth that were needed in the regular setup of the course.

The data in the tables (Tables 1, 2, 3, 4, 5, 6, 7) are mainly descriptive. To improve readability and for a quick view, the ranking with regard to the Likert scales was provided as mean values, 25th and 75th percentiles and the according interquartile ranges (IQR). Additionally, whenever useful, statistical analysis is also added.

**TABLE 1:** Students' evaluation of the didactic quality of the course and the staff (Figure 6: Section 2) with mean, percentiles and IQR (=interquartile range).

2 evaluation didactic quality		[1] strongly disagree		[2] disagree		[3] neutral		[4] agree		[5] strongly agree		[−] no specification		Mean; [25th; 75th percentile]; IQR
		*n*	[%]	*n*	[%]	*n*	[%]	*n*	[%]	*n*	[%]	*n*	[%]	
Q1	Teaching staff expertise	0	0.0	0	0.0	2	4.5	10	22.7	30	68.2	2	4.5	5 [4;5]; 1
Q2	Satisfactory staff assistance/support	0	0.0	1	2.3	4	9.1	23	52.3	16	36.4	0	0.0	4 [4;5]; 1
Q3	Satisfactory supervision	1	2.3	1	2.3	8	18.2	19	43.2	15	34.1	0	0.0	4 [4;5]; 1
Q4	Course organisation	1	2.3	4	9.1	9	20.5	20	45.5	10	22.7	0	0.0	4 [3;4]; 1
Q5	Practicing opportunities	12	27.3	18	40.9	10	22.7	2	4.5	2	4.5	0	0.0	2 [1;3]; 2

### 3.2 Didactic Quality of the Course

The entire course (staff, supervising, organisation) mainly led to a positive rating (> 70%). Only very few (11.4%) were not satisfied with the organisation. Despite the overwhelming positive ratings, more than 2/3 (=68%) of the students complained about not having enough time for all treatment steps (Table 1).

### 3.3 Students' Perception About the Diagnostic Part

Clinical findings matched in all sections (endodontics, periodontology and restorative dentistry) excellently and led to agreement of the students (86.46%–90.9%) (Table 2). The medical history was comprehensible and helped in the diagnosis and decision‐making process (93.2%–95.5%).

**TABLE 2:** Students' evaluation concerning the suitability of the radiographic findings and the medical history (Figure 6: Section 3) with mean, percentiles and IQR (=interquartile range).

3 diagnosis		[1] strongly disagree		[2] disagree		[3] neutral		[4] agree		[5] strongly agree		[−] no specification		Mean; [25th; 75th percentile]; IQR	*p*
		*n*	[%]	*n*	[%]	*n*	[%]	*n*	[%]	*n*	[%]	*n*	[%]		
Q1	Matching radiographic findings endo	0	0.0	0	0.0	4	9.1	14	31.8	26	59.1	0	0.0	5 [4;5]; 1	*p* = 0.96
Q2	Matching radiographic findings perio	0	0.0	0	0.0	3	6.8	13	29.5	26	59.1	2	4.5	5 [4;5]; 1	
Q3	Matching radiographic findings resto	0	0.0	0	0.0	4	9.1	9	20.5	29	65.9	2	4.5	5 [4;5]; 1	
Q4	Medical history	0	0.0	0	0.0	3	6.8	7	15.9	34	77.3	0	0.0	5 [4.5;5]; 0.5	
Q5	Medical history and diagnosis	0	0.0	0	0.0	2	4.5	15	34.1	27	61.4	0	0.0	5 [4;5]; 1	
Q6	Decision making	0	0.0	0	0.0	3	6.8	15	34.1	26	59.1	0	0.0	5 [4;5]; 1	

### 3.4 Students' Perception About the Model and Its Endodontic, Periodontal and Restorative Treatments (Tables 3, 4, 5)

**TABLE 3:** Students' rating concerning the endodontic treatment (Figure 6: Section 4) with mean, percentiles and IQR (=interquartile range).

4 endodontics^a^		[1] favouring 3D‐model		[2] identical		[3] favouring conventional model/approach/real teeth		Mean; [25th; 75th percentile]; IQR
3D‐print model vs. conventional		*n*	[%]	*n*	[%]	*n*	[%]	
Q1	Teeth hard/soft	4	9.3	1	2.3	38	88.4	3 [3;3]; 0
Q2	Radiopacity	4	9.3	30	69.8	9	20.9	2 [2;2]; 0
Q3	Tightness root canals	8	18.6	20	46.5	15	34.9	2 [2;3]; 1
Q4	Root canal preparation	3	7.0	18	41.9	22	51.2	3 [2;3]; 1
Q5	Gauging procedure	4	9.3	26	60.5	13	30.2	2 [2;3]; 1
Q6	Root canal filling	4	9.3	32	74.4	7	16.3	2 [2;2]; 0
Q7	x‐ray	4	9.3	19	44.2	20	46.5	2 [2;3]; 1
Q8	Suitabilizty clinical practice	23	53.5	15	34.9	5	11.6	1 [1;2]; 1
Q9	Exam fairness	18	41.9	7	16.3	18	41.9	2 [1;3]; 2
Q10	Suitability practical exam	18	41.9	7	16.3	18	41.9	2 [1;3]; 2
Q11	Overall handling	5	11.6	10	23.3	28	65.1	3 [2;3]; 1

		Yes		No				
		*n*	[%]	*n*	[%]			
Q12	Comprehensible anatomy	36	83.7	7	16.3			
Q13	Realistic tactility	29	67.4	14	32.6			

^a^ *n* = 43; one student did not finish endodontic treatment (no rating).

**TABLE 4:** Students' rating concerning restorative treatment (Figure 6: Section 5) with mean, percentiles and IQR (=interquartile range).

5 restorative		[1] favouring 3D‐model		[2] identical		[3] favouring conventional model/approach/real teeth		Mean; [25th; 75th percentile]; IQR
3D‐print model vs. conventional		*n*	[%]	*n*	[%]	*n*	[%]	
Q1	Decay hardness	1	2.3	3	6.8	40	90.9	3 [3;3]; 0
Q2	Enamel hardness	0	0.0	3	6.8	41	93.2	3 [3;3]; 0
Q3	Dentin hardness	1	2.3	1	2.3	42	95.5	3 [3;3]; 0
Q4	Preparation	16	36.4	12	27.3	6	13.6	2 [1;2]; 1
Q5	Placing matrices	13	29.5	8	18.2	23	52.3	3 [1;3]; 2
Q6	Fill cavities	10	22.7	32	72.7	2	4.5	2 [2;2]; 0
Q7	Polishing	8	18.2	20	45.5	16	36.4	2 [2;3]; 1
Q8	Suitability clinical practice	14	31.8	15	34.1	15	31.6	2 [1;3]; 2
Q9	Exam fairness	9	20.5	19	43.2	16	36.4	2 [2;3]; 1
Q10	Suitabililty practical exams	12	27.3	17	38.6	15	34.1	2 [1;3]; 2
Q11	Overall handling	12	27.3	12	27.3	20	45.5	2 [1;3]; 2

		Yes		No				
		*n*	[%]	*n*	[%]			
Q12	Comprehensible anatomy	40	90.9	4	9.1			
Q13	Realistic tactility	27	61.4	17	38.6			

**TABLE 5:** Students' rating concerning periodontal treatment (Figure 6: Section 6) with mean, percentiles and IQR (=interquartile range).

6 periodontics		[1] favouring 3D‐model		[2] identical		[3] favouring conventional model/approach		Mean; [25th; 75th percentile]; IQR
3D‐print model vs. conventional		*n*	[%]	*n*	[%]	*n*	[%]	
Q1	Probing	12	27.3	25	56.8	7	15.9	2 [1;2]; 1
Q2	Recession measurement	9	20.5	29	65.9	6	13.6	2 [2;2]; 0
Q3	Gingiva hardness	32	72.2	3	6.8	9	20.5	1 [1;2]; 1
Q4	Hyperplasia measurement	10	22.7	28	63.6	6	13.6	2 [2;2]; 0
Q5	Bone defects	8	18.2	26	59.1	10	22.7	2 [2;2]; 0
Q6	Performance handscaling	15	34.1	8	18.2	21	47.7	2 [1;3]; 2
Q7	Performance sonic scaling	13	29.5	15	34.1	16	36.4	2 [1;3]; 2
Q8	Suitability for practice	9	20.5	16	36.4	19	43.2	2 [2;3]; 1
Q9	Exam fairness	8	18.2	15	34.1	21	47.7	2 [2;3]; 1
Q10	Suitability practical exams	13	29.5	8	18.2	23	52.3	3 [1;3]; 2
Q11	Overall handling	7	15.9	13	29.5	24	54.5	3 [2;3]; 1

		Yes		No				
		*n*	[%]	*n*	[%]			
Q12	Comprehensible anatomy	42	95.5	2	4.5			
Q13	Realistic tactility	31	70.5	13	29.5			

The 3D‐printed model perfectly simulated anatomical (83.7%–95.5%) aspects, but tactility was evaluated significantly poorer compared to real teeth or the regular commercially available models (Frasaco ANA 4 and A‐PB OC; Frasaco, Tettnang, Germany (less than 70% positive ratings)).

Despite the predominant more difficult handling of the 3D‐printed model compared to the conventional models independent of the subject area, the different parts of the treatment (endodontic, periodontal, restorative) revealed heterogeneous results regarding the suitability for practice and examinations (questions 8–11 in the corresponding sections). While more than half of the students rated the printed teeth positively for endodontic practice, the students were undecided regarding restorative treatment and predominantly negative regarding periodontal treatment.

In all categories (question 4.11; 5.11, 6.11), students' rated handling of the 3D‐printed model as more difficult compared to the conventional approach. Thus, treatment was more demanding in the complete setting obtained.

### 3.5 Students' Perception About the Estimated Learning Outcome (Tables 6 and 7)

**TABLE 6:** Self‐assessment of learning outcome and of obtained confidence (Figure 6: Section 7) with mean, percentiles and IQR (=interquartile range). Learning outcome and confidence level in the different disciplines were statistically analysed (Chi‐square test).

7 learning outcome		[1] strongly disagree		[2] disagree		[3] neutral		[4] agree		[5] strongly agree		[−] no specification		Mean; [25th; 75th percentile]; IQR	*p*
		*n*	[%]	*n*	[%]	*n*	[%]	*n*	[%]	*n*	[%]	*n*	[%]		
Q1	Learning success general	0	0.0	3	6.8	13	29.5	18	40.9	10	22.7	0	0.0	4 [3;4]; 1	
Q2	Endo compared to regular	2	4.5	8	18.2	16	36.4	9	20.5	2	4.5	7	15.9	3 [2;4]; 2	
Q3	Perio compared to regular	5	11.4	9	20.5	14	31.8	12	27.3	2	4.5	2	4.5	3 [2;4]; 2	*p* = 0.002
Q4	Resto compared to regular	0	0.0	3	6.8	10	22.7	23	52.3	7	15.9	1	2.3	4 [3;4]; 1	
Q5	Comparability 3D‐Printed teeth to real teeth	12	27.3	18	40.9	10	22.7	4	9.1	0	0.0	0	0.0	2 [1;3]; 2	
Q6	Confidence endo	0	0.0	6	13.6	10	22.7	20	45.5	6	13.6	2	4.5	4 [3;4]; 1	
Q7	Confidence perio	3	6.8	7	15.9	9	20.5	20	45.5	6	13.6	0	0.0	3 [3;4]; 1	*p* = 0.60
Q8	Confidence resto	2	4.5	2	4.5	8	18.2	25	56.8	7	15.9	0	0.0	3 [3;4]; 1	

**TABLE 7:** Effects of training with the interdisciplinary 3D‐printed model in terms of enthusiasm and skills (Figure 6: Section 8) with mean, percentiles and IQR (=interquartile range).

8 effects of training		[1] strongly disagree		[2] disagree		[3] neutral		[4] agree		[5] strongly agree		[−] no specification		Mean; [25th; 75th percentile]; IQR	*p*
		*n*	[%]	*n*	[%]	*n*	[%]	*n*	[%]	*n*	[%]	*n*	[%]		
Q1	Endodontics enthusiasm	0	0.0	4	9.1	4	9.1	12	27.3	21	47.7	2	4.5	5 [4;5]; 1	
Q3	Perio enthusiasm	2	4.5	13	29.5	8	18.2	12	27.3	9	20.5	0	0.0	3 [2;4]; 2	*p* = 0.0005
Q5	Restorative enthusiasm	0	0.0	2	4.5	2	4.5	21	47.7	19	43.2	0	0.0	4 [4;5]; 1	
Q2	Root canal skills	0	0.0	4	9.1	8	18.2	22	50.0	8	18.2	2	0.0	4 [3;4]; 1	
Q4	Scaling skills	3	6.8	8	18.2	13	29.5	11	25.0	9	20.5	0	0.0	3 [2.5;4]; 1.5	*p* = 0.008
Q6	Resto skills	0	0.0	4	9.1	4	9.1	21	47.7	15	34.1	0	0.0	4 [4;5]; 1	

*Note:* Enthusiasm and valued skills were analysed with regard to the differences between the various disciplines. (Note: The order of the questions was changed compared to the original questionnaire for better readability and visibility of the table/analysis).

The estimated learning outcome due to the use of the new model was significantly better in restorative dentistry compared to endodontics and periodontics (*p* = 0.002), but practicing did not lead to different confidence levels in the three disciplines (*p* > 0.05). Enthusiasm during performance of periodontal treatment was significantly reduced compared to the other two sections (*p* = 0.0005). Thus, the 3D‐printed model led to subjective better self‐estimated skills in endodontic and restorative compared to periodontal treatment (*p* < 0.05).

### 3.6 Free Comments

The answers in the free comments of the students were screened, categorised and weighed by the authors. In general, the positive votes outweighed the negative ones, but the students also had some aspects that should be improved in future. The following aspects to improve the model were mentioned most frequently:

The problems that occurred were either model or case‐specific aspects. The fixation of the teeth achieved by the patrix‐matrix design revealed more mobility compared to the conventional models—fixation should be improved. Additionally, teeth should be harder. Furthermore, the students complained about the gingival hyperplasia that hampered the complete periodontal treatment (=case specific problem).

Apart from that, a vast majority of the students highlighted the excellent clinical relevance by performing a complete treatment. Instead of performing standardised tasks in each discipline, the interdisciplinary approach with all sub‐disciplines was particularly praised. The most reported benefit was that the 3D‐printed model allowed a transfer performance to manage a ‘real’ case/patient with the aim of performing a complete treatment based on an anamnesis, clinical examination and interpretation of provided radiographs by developing an individual treatment plan. Students mentioned, that is, that the medical history and concomitant dental findings led to a better understanding of the complexity and the necessity of pharmacological knowledge. Medication of patients may have a huge impact on dental treatment.

Additionally, during examination, all students must perform identical treatments at the same difficulty level, allowing a better comparability of the performances to ensure that the best possible fairness is guaranteed.

## 4 Discussion

The development of 3D‐printed models in dental education has been a major advancement in recent years and offers potential for further development [31, 32]. In the field of dental education, new digital tools have emerged in recent years for theoretical teaching and practical training, as well as different modalities for virtual interaction with students or testing their skills [10, 29, 33, 34]. Digital training may improve students' 3D visualisation skills and helps to better understand and assimilate the required specific didactic information [35, 36]. Students strongly recommended in future education the implementation of the newly developed 3D‐printed teaching model that for the first time offered an interdisciplinary approach and a complete case scenario with all essential treatment steps (diagnosis, treatment planning and execution). Thus, the null‐hypothesis was rejected as the 3D‐printed models may represent a suited way to enhance students' (cognitive) skills and self‐confidence, and consequently may improve patient care. Furthermore, this is claimed to be helpful for a professional career [37].

As an alternative to 3D‐printed models, virtual learning environments, such as the Simodont (Moog, Nieuw‐Vennep, the Netherlands), can replicate many scenarios and may serve as haptic dental trainers [38]. While such systems can be very useful for performing manual dexterity exercises [39], essential aspects such as clinical diagnostics or direct restorative therapy cannot be performed virtually.

Recent studies only focused on printed models for particular dental procedures; for example, endodontic treatment [29, 30, 40], dental implant placement [41], surgical treatment [26] and dental traumatology [28]. Only a few studies dealt with a more complex approach including prosthodontic and restorative treatment [10, 13, 15], and implantology [42] but did not provide the additional patient history with the associated concomitant diseases or a simulated emergency situation. Thus, this was the very first approach to implement a complete clinical case into undergraduate teaching that included a diagnosis process based on medical and dental history as well as radiographic findings in order to improve diagnosis competence and practical skills.

Usually, training using standardised models for purchase is widespread, but it does not allow for much modification and variability. Especially when measuring the periodontal status, it may be categorised as a dull memorising process when the same model is used year by year. This does not represent the true skills of the students. Individual models with changing clinical conditions may be better suited in terms of any kind of bias. The present model is a modular model and therefore allows minor and major modifications using the corresponding software prior to the printing process.

In the present study, undergraduate students (third year) who just finished the undergraduate clinical training (phantom‐)course in restorative dentistry evaluated the 3D‐printed universal teaching model. Consequently, they had adequate knowledge and skills in all restorative treatment procedures, endodontics and periodontology that were necessary for a successful treatment of the provided case. The students' perception concerning the clinical findings was extraordinary. Thus, 3D‐printing allows to guarantee anatomical accuracy and implements individual findings that can even be modified when using a modular approach [21, 29].

The significantly poorer ratings of the participants concerning the periodontal treatment are partly comprehensible due to the medication‐induced gingival hyperplasia that hampers the determination of pocket depths and also the consecutive scaling and root planning. When compared to the conventional models, the handling was generally rated as more demanding (question 11 in each section). This may be attributed to model‐specific aspects and the complexity of the simulated case that combined multiple disease patterns. Especially hard tissues such as enamel and dentine lack hardness (i.e., Q 4.1–4.4 and 5.1–5.4) and do not represent identical haptics compared to real teeth [43].

Nonetheless, 3D‐printed teeth are claimed to be equally suited for preclinical educational purposes [44]. Apart from that, individual 3D‐printed models—presenting complete case scenarios—obviously enhanced enthusiasm and led to more confidence with regard to real clinical situations.

3D printed teeth/models can improve the teaching of dental students in several ways:

1. Hands‐on practice: 3D‐printed models can provide dental students with a realistic model to practice their skills. These models can be used to simulate a wide range of dental/interdisciplinary procedures, from simple fillings to even complex treatments. Students can work on these models repeatedly, allowing them to refine their skills before working on real patients [45, 46, 47].
2. Anatomical accuracy: 3D‐printed teeth/models can be produced with a high degree of anatomical accuracy. The exact shape and structure of teeth, including any irregularities or variations, may be simulated. This level of detail is difficult to achieve with traditional dental models and can help students to develop a better understanding of dental anatomy [43, 48].
3. Customization: 3D‐printing technology allows the creation of customised dental models based on the needs of individual students. This level of customisation can be particularly useful for advanced dental procedures that require a high degree of precision [21, 29].
4. Cost‐effectiveness: 3D‐printing technology can be used to produce dental models at lower costs than traditional methods, especially when used in a modular approach [34]. Due to the developments of the 3D‐printing technique, the models may even get more affordable in the future. This can make it easier for dental schools to provide students with the tools they need to learn and practise effectively.

By allowing students to practice their skills on 3D‐printed models, even in combination with virtual reality and augmented reality approaches, dental curricula can better prepare them for daily clinical scenarios and may improve the quality of care they provide to patients [36, 49].

### 4.1 Limitations and Perspectives

The study focused on dental students from the same faculty. Thus, there is an immanent risk of selection bias, homogeneity of the sample and a social desirability bias, which could affect the generalisability of the findings. This fact applies to all studies that are based on an evaluation of a university's coherence and must therefore be mentioned.

However, another potential bias is negligible because the results of this specific educational program had no impact on passing this course of 3rd year students. The selection of the cohort was random because students were taught until this project in accordance with the existing national catalogue of learning objectives. The student profile/mix of each semester cannot be influenced in advance. The aim was to give the students the possibility to perform all treatment steps in a universal teaching model and not with numerous specific training models exclusively for periodontology, restorative tasks, or endodontics. The students were able to plan and carry out their interdisciplinary treatment steps freely and without any time pressure. Nonetheless, a bias could be assumed since the students had to deal with the same case individually and they had to carry out the therapy after appropriate consultation with their supervisor because an exchange between the students after the individual case presentation could not be ruled out completely. However, the students were instructed not to discuss their individual treatment plans.

Apart from that, with the introduction of such innovative models and/or technologies, more opportunities could arise: Academic teaching concepts can be transformed into a dynamic learning and teaching system that allows students to interact with virtual or simulated patients, make diagnoses and intervene when necessary [37]. Additionally, personalised learning plans with individual programmes matching to the individual paces may be feasible, enabling reflection and self‐feedback of students without having to rely on experienced teachers [37]. Students can advance stepwise and it may be possible to access severe and complex cases without a negative consequence for a real patient.

Future studies should be performed in a multi‐centric design with a much larger group of students to reduce possible biases and to compare the student skills and the according teaching contents at different universities. Another aspect would be the evaluation of the influence on the quality of patient care. However, this would have to be done in prospective studies that are free of any observational bias by persons involved in this teaching project. Furthermore, as a desirable side effect, university networking and case sharing would exponentially increase the number of treatment options and could significantly reduce the potential workload of teaching staff.

Case‐ and model‐specific aspects were a limitation, too, as this was a preliminary study to assess the impact of implementing individual 3D‐printed interdisciplinary models in dental education. The study should be repeated with (i) a larger number of students, (ii) in following semesters to minimise cohort‐specific aspects, (iii) implemented improvements concerning the fixation of the teeth and (iv) with other interdisciplinary cases.

An optimization of patrix‐matrix system could improve the fixation or the sextants could be produced in two parts and fixed with screws to achieve a non‐detachable anchoring by means of a splint. Alternatively, the model could be printed as a single work peace with secondary detachable connections, for evaluation purposes. Additionally, the properties of the gum mask still need to be improved in order to increase the durability of the models and to reduce costs in future. The changes should always be accompanied by repeating the questionnaire for continuing optimization of dental teaching models. The teaching content and also the 3D‐printed models should be constantly adapted to the developments in the ageing society and the corresponding needs of the patients. By means of the additional individualisation through the exchange of sextants a variety of cases are possible in the long term.

From a technical point of view, the rapid development in 3D printing should also be mentioned, because with the possibility of multi‐colour printing, it will be possible to print such models in one piece, which would save time and costly assembly of the individual components. This would possibly also extend the evaluation options, as it could, for example, enable both quantitative and qualitative cleaning during periodontal treatment when removing calculus by printing the calculus adherently onto the root surfaces.

## 5 Conclusion

Individual, interdisciplinary 3D‐printed teaching models that cover complex treatment strategies may be well suited to improve the skills of dental students and favour their enthusiasm due to the integrated diagnosis and treatment planning process. Those models may allow avoiding memorising biases from using the same models year by year. Managing complete cases prepares well for the clinical situation and helps students feel confident in the future, and even allows achieving diagnosis competence. In addition, improved comparability of students' competence levels due to exactly the same requirements and thus better fairness in examination situations is ensured. However, some crucial aspects of natural teeth and model‐specific weaknesses are not yet perfectly addressed and need to be improved in the future.

## Author Contributions

All authors participated sufficiently in the work to take public responsibility for appropriate portions of the content and agreed to be accountable for all aspects of the work in ensuring that questions related to the accuracy or integrity of any part of the work are appropriately investigated and resolved.

## Consent

Informed consent was obtained from all subjects involved in the study.

## Conflicts of Interest

The study was funded by the Ministry of Innovation, Science and Research of the State of North Rhine‐Westphalia (Act for the Improvement of the Quality of Teaching and Academic Programs at North Rhine‐Westphalian Universities (Study Quality Act)).

## Data Availability

The data that support the findings of this study are available from the corresponding authors upon reasonable request.
